# Yin and Yang of disease genes and death genes between reciprocally scale-free biological networks

**DOI:** 10.1093/nar/gkt683

**Published:** 2013-08-09

**Authors:** Hyun Wook Han, Jung Hun Ohn, Jisook Moon, Ju Han Kim

**Affiliations:** ^1^Division of Biomedical Informatics, Seoul National University Biomedical Informatics (SNUBI), Seoul National University College of Medicine, Seoul 110799, Korea, ^2^College of Medicine, CHA General Hospital, CHA University, Seoul 135081, Korea and ^3^Systems Biomedical Informatics Research Center, Seoul National University, Seoul 110799, Korea

## Abstract

Biological networks often show a scale-free topology with node degree following a power-law distribution. Lethal genes tend to form functional hubs, whereas non-lethal disease genes are located at the periphery. Uni-dimensional analyses, however, are flawed. We created and investigated two distinct scale-free networks; a protein–protein interaction (PPI) and a perturbation sensitivity network (PSN). The hubs of both networks exhibit a low molecular evolutionary rate (*P* < 8 × 10^−12^, *P* < 2 × 10^−4^) and a high codon adaptation index (*P* < 2 × 10^−16^, *P* < 2 × 10^−8^), indicating that both hubs have been shaped under high evolutionary selective pressure. Moreover, the topologies of PPI and PSN are inversely proportional: hubs of PPI tend to be located at the periphery of PSN and vice versa. PPI hubs are highly enriched with lethal genes but not with disease genes, whereas PSN hubs are highly enriched with disease genes and drug targets but not with lethal genes. PPI hub genes are enriched with essential cellular processes, but PSN hub genes are enriched with environmental interaction processes, having more TATA boxes and transcription factor binding sites. It is concluded that biological systems may balance internal growth signaling and external stress signaling by unifying the two opposite scale-free networks that are seemingly opposite to each other but work in concert between death and disease.

## INTRODUCTION

Biological systems are often described as complex networks whose vertex connectivities follow a scale-free power-law distribution (1). Complex interactions of biological building blocks, such as genes, proteins and metabolites, have been modeled with network graphs, such as protein interaction, metabolic, signaling and transcriptional regulatory networks. The scale-free topology of a network may arise from ‘network growth’ and ‘preferential attachment’, endowing the network with robustness against random errors owing to its relatively small number of functional hubs and many peripheral nodes. The so-called attack vulnerability, however, may also arise when a few key hubs are precisely perturbed, splitting the network into several smaller pieces (1,2).

Living cells finely balance internal growth signaling with external stress signaling. Biologically, robustness against random errors and attack vulnerability are associated with the lethality of mutations in the protein–protein interaction (PPI) network. Deleterious mutations of highly connected hub proteins in the PPI network of yeast are three times more likely to be lethal than those of less connected peripheral ones (3). Therefore, the scale-free topology of PPI network seems to have been shaped by evolutionary selection pressure, enabling organisms to be robust against random mutations and lethal only from disruptions of a few critical proteins with many interacting partners. Each gene’s contribution to sustain internal growth or ‘essentiality’ is well represented by the connectivities of protein interactome.

Meanwhile, with the advent of large-scale gene expression profiling technologies like microarrays, expression variability in different biological conditions has emerged as an inherent trait of a gene. Expression variability is reported to be associated with genomic and epigenomic architectures. Genes with highly variable expression patterns are more likely to have a TATA box and more upstream regulatory elements and chromatin regulators than those with less expression variability (4,5). Recently, the view that genes with high expression variability or noisy expression are stress-responsive genes, expressed in tune with physiological needs, conferring resistance to environmental perturbations, is gaining acceptance (6,7).

Expression variability in response to environmental perturbations, especially genetic perturbations, has been modeled with the so-called ‘perturbation sensitivity network (PSN)’ or ‘disruption network’ (8,9). The PSN is a ‘directed bipartite’ graph between a ‘gene set’ and a ‘mutation set’ where an edge connects a deletion mutant to a gene when the gene is significantly up- or downregulated by the deletion mutant (8). Because a deletion mutant is also a gene, the PSN can also be simply viewed as a directed graph of genes. The perturbation sensitivity is defined as the in-degree of genes in the bipartite graph, reflecting the extent to which the transcription of each gene is significantly affected by random genetic perturbations (or deletion mutations). Hub genes of the PSN or highly perturbation-sensitive genes are non-essential and involved in biological processes like ‘interaction with cellular environment’ and ‘metabolism’. Core genes of the perturbation network code for the less-connected proteins in the PPI network and are located in the periphery of the protein interactome (8).

Each biological network usually models one aspect of biological phenomena, like the physical binding relationship between proteins, the transcriptional regulation between transcription factors and target genes or the chemical reaction between enzymes and metabolites. The finding that perturbation network cores are located in the periphery of the protein interaction network motivated us to explore the architecture of the combined network representing seemingly unrelated biological properties, physical interaction among proteins and transcriptional responsiveness to genetic perturbations.

We built a yeast PPI network from physical interaction data in the *Saccharomyces* genome database (http://www.yeastgenome.org) and a yeast PSN network from a genome-wide transcriptional profiling study of 276 gene deletion mutant strains of *Saccharomyces cerevisiae* (10). The two networks differ from each other in that the PPI network models the physical interactions between proteins, whereas the PSN network represents the transcriptional response of each gene to random genetic perturbations.

In the present study, we investigated not only network topology, evolutionary pressure, distribution of classified genes (essential, disease, drug-target and TATA-containing genes), transcription factor binding site (TFBS) and functional enrichment but also the interrelation of the two networks, PPI and PSN. Finally, based on the results, we propose an improved network model of biological systems.

## MATERIALS AND METHODS

**Construction of PPI.** Protein interaction data were retrieved from the yeast genome database (http://www.yeastgenome.org) and then filtered only for physical interactions. The data consist of 5531 genes, with *K*_PPI_ degrees ranging from 1 to 2569 (Figure 2A).

**Construction of PSN.** PSN was constructed from the mRNA expression profiling of 6326 yeast ORFs in 276 gene deletion mutant strains, excluding drug treatments in *S. cerevisiae* (10). A PSN network is a directed bipartite graph where one vertex set contains significantly up- or downregulated genes in deletion mutants, which constitute the other vertex set. A directed link is made from deletion mutant *j* to gene *i* if the expression of gene *i* is significantly altered in the deletion mutant *j*. Based on the ‘error model’ correcting for each gene measurement error and biological noise, *P*-value was assigned for each pair of gene and deletion mutant (10). In all, 4280 genes showed significant expression changes in more than one deletion mutant, and 212 deletion mutants affected more than one gene (*P* < 0.01). Finally, we corrected non-standard names with standard names and removed merged or deleted ORFs in the dataset by referencing the *Saccharomyces* genome database (http://www.yeastgenome.org). A matrix of 4226 genes and 212 deletion mutants, *D = <d_ij_>*, were created for gene *i* and deletion mutant *j* where:

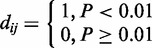



Perturbation sensitivity, *S_i_*, of gene *i* is defined as the in-degree of the gene *i* in the directed bipartite graph, reflecting the responsiveness of gene *i* to external perturbations. It can also be denoted as *K*_PSN_, and the range was from 1 to 49.

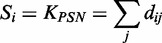

***m*-core.** An *m*-core of a graph *G* is defined as a maximal connected subgraph of *G* in which all vertices have a degree of at least *m* (8). Equivalently, it is one of the connected components of the subgraph of *G* formed by repeatedly deleting all vertices of a degree less than *m*. One can iteratively apply this ‘peeling’ procedure by increasing *m* to produce subgraphs with more hub vertices of *G*. Likewise, *m*-core_PPI_ or *m*-core_PSN_ is defined as a maximal connected subgraph of a PPI or a PSN network, respectively, in which all genes have a degree *K*_PPI_ or *K*_PSN_ of at least *m*.

**Excess retention.** ‘Excess retention’ was proposed by Wuchty *et al.* (11). It is defined as the degree to which genes with a certain property *A* is over- or under-represented in ‘*m*-core’ compared with that in the whole gene groups, i.e. *1*-core. The fraction of genes with property *A* in the whole group with *N* genes is
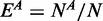
. If *m*-core contains
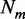
genes and the number of genes with property
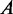
in *m*-core is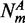, then the excess retention of the genes with property *A* in *m*-core is given by:

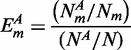



**Enrichment of functional categories.** We performed enrichment analysis of functional categories for each of the node-degree groups of *K*_PPI_ and *K*_PSN_. The MIPS database (http://mips.gsf.de) contains functional catalogs and is made of a functional annotation scheme for systematic classification of proteins from the whole genome and provides online tools (http://mips.gsf.de/proj/funcatDB) for statistical testing of functional enrichment by using hypergeometric distribution. Enrichment *P*-values for each functional category of gene groups were corrected for multiple hypothesis testing by calculating false discovery rate (FDR)-adjusted *P*-values (<0.01).

**Network visualization.** There were a few double-deletion mutant strains that we excluded from the dataset for the purpose of PSN network visualization. PPI and PSN networks have 3679 nodes with 26 088 edges and 3392 nodes with 9820 edges, respectively. The joint network has 3756 nodes with 35 908 edges of which 98 edges are in common in the joint network. We used the ‘igraph’ library (http://igraph.sourceforge.net) in the R statistical package for network visualization and applied the ‘Layout.frutcherman.reingold’ function of the library for three-dimensional network visualization.

**dN/dS (evolutionary rate).** dN/dS is the ratio of the number of non-synonymous substitutions per non-synonymous site (dN) to the number of synonymous substitutions per synonymous site (dS), which can be used as an indicator of selective pressure acting on a protein-coding gene (12,13). We obtained 3036 adjusted dN/dS values as evolutionary rate indicators from a previously published dataset using four-way yeast species alignments for *S. cerevisiae* genes (12). We were able to obtain adjusted dN/dS values for 3006 of 5981 yeast genes (range from 0 to 0.523705) including 2947 of 5531 PPI and 2126 of 4226 PSN genes. Of the 3776 genes participating in both networks, 2067 genes have adjusted dN/dS values.

**Codon adaption index (CAI).** Codon usage bias refers to differences in the frequency of occurrence of synonymous codons in coding DNA. The overabundance in the number of codons allows many amino acids to be encoded by more than one codon. Codon usage bias is generally known to reflect a balance between mutational biases and natural selection for translational optimization. Codon adaption index (CAI) is one of the general measures for codon usage bias. It is measured based on the similarity of codon usage between a given gene and highly expressed genes from a given organism (14–16)*.* We retrieved precalculated CAIs for 6623 yeast genes from the *Saccharomyces* genome database (http://www.yeastgenome.org). We were able to obtain CAI values for 5903 of 5981 yeast genes (range from 0 to 0.924), including 5454 of 5531 PPI and 4200 of 4226 PSN genes. Of 3776 genes participating in both networks, 3751 genes have CAI values.

**Human homolog genes.** In all, 1498 human homologs of 5981 yeast genes were retrieved from the HomoloGene (http://www.ncbi.nlm.nih.gov/homologene) database.

**Lethal (essential) genes.** Lethality information of 5448 of 5981 yeast genes was obtained from the MIPS Comprehensive Yeast Genome Database (http://mips.helmholtz-muenchen.de/genre/proj/yeast/). For the 1498 human homologs of yeast genes, lethality information was available for 1492 yeast genes.

**Disease genes.** A total of 1498 yeast homologs were searched against the Online Mendelian Inheritance in Man (OMIM, http://www.ncbi.nlm.nih.gov/omim) database. OMIM is a database of human disease phenotypes and causal genetic mutations, mainly containing genetic disorders with Mendelian inheritance (17). We obtained 285 OMIM disease genes and 1213 non-disease genes.

**Drug target genes**. Information on drug target of 1498 human homologs of yeast genes was obtained from the DrugBank (http://www.drugbank.ca) database.

**Number of TFBSs.** We retrieved TFBS from the upstream sequence of yeast genes from the YEASTRACT database (http://www.yeastract.com). The number of TFBSs per yeast gene ranged from 0 to 58.

**TATA-containing genes.** TATA-box information of 5541 of 5981 yeast genes was obtained from the raw data of Basehoar *et al*. (18).

## RESULTS

### Network topology analysis of PPI and PSN

**Figure 1.**
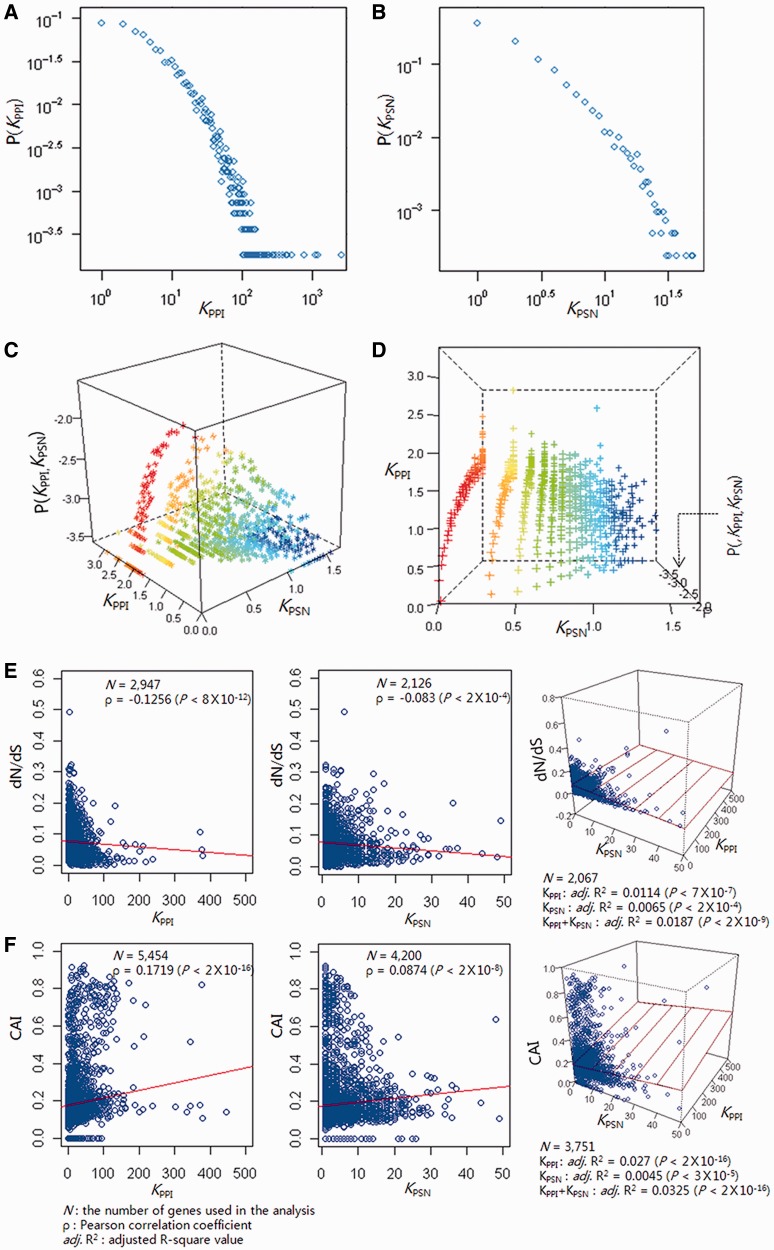
Degree distribution and evolutionary conservation within biological networks. The (**A**) protein interaction and (**B**) perturbation sensitivity networks are both scale-free with approximate exponents of 2.48 (γ_PPI_) and 2.24 (γ_PSN_), respectively. (**C** and **D**) The three-dimensional plots demonstrate that the two distinct networks are reciprocally intertwined to constitute a curved surface. Nodes are coded with different colors according to node degrees (*K*_PPI_ and *K*_PSN_). Scatter plots between the node degrees (*K*_PPI_ and *K*_PSN_) and (**E**) the protein evolutionary rate (dN/dS) and (**F**) the CAI demonstrate that hubs of both networks are under high evolutionary selection pressure.

According to the degree-distribution plots, both the PPI and the PSN are scale-free with approximate exponents of 2.48 (γ_PPI_) and 2.24 (γ_PSN_), respectively (Figure 1A and B). The scale-freeness of the joint network is further explored. Figure 1C exhibits the three-dimensional degree-distribution plot where the vertical axis depicts log-transformed joint-degree distribution and the horizontal axes depict log-transformed degrees in each network. Figure 1D visualizes the same plot in a different direction. The three-dimensional degree-distribution plot (Figure 1C and D) can be fit with a regression plane, which we call ‘high dimensional scale-free’. Interestingly, distribution of coordinates in the horizontal plane in Figure 1C (or coronal plane in Figure 1D) demonstrates that the degrees in each network, i.e. *K*_PPI_ and *K*_PSN_, are inversely proportional or ‘reciprocal’ (Pearson correlation coefficient, −0.039 with *P*-value = 0.0028). Genes highly connected in the protein interaction network are least likely to be hubs in the perturbation network, and vice versa*.*

### Evolutionary analysis of PPI and PSN

Hubs of PPI and PSN are both important genes for the fitness of the organism, as they show a low protein evolutionary rate and a high codon adaptation index (Figure 1E and F). In the ‘neutral’ theory of molecular evolution (19), evolutionary changes at the molecular level are caused by drift and fixation of random mutations that do not affect the fitness of the organism. Hence, the rate of evolution should be lower in proteins whose deletions affect the survival of the organism (20).

Protein evolutionary rate (dN/dS) is defined as the ratio of non-synonymous divergence (dN) to synonymous divergence (dS) (12). As previously reported (21), PPI hubs evolve slowly; *K*_PPI_ is negatively correlated with dN/dS (*P* < 8 × 10^−^^12^). PSN hubs also evolve slowly; *K*_PSN_ is negatively correlated with dN/dS (*P* < 2 × 10^−^^4^). Hubs of both PPI and PSN have evolved slowly and their sequence divergences are constrained under high evolutionary selection pressure, which suggests that they are important genes that are crucial for the survival of the organism.

Another indirect measure of evolutionary selection pressure is the synonymous codon usage bias. Synonymous codons for an amino acid are not used randomly. It is experimentally confirmed that mRNAs made up of preferred codons are translated faster than those artificially modified to have rare codons (22). This codon preference is the balance between mutational bias and translational efficiency, reflecting selection pressure for translational optimization. The CAI is a measure of synonymous codon usage bias, and important genes have high CAI, as their efficient translation may confer a survival benefit (14). Both *K*_PPI_ and *K*_PSN_ are positively correlated with CAI values (*P* < 2 × 10^−^^16^ and *P* < 2 × 10^−^^8^, respectively); high evolutionary selection pressure seems to act on both hubs of PPI and PSN, reflecting their important role in the fitness of the organism.

### Lethal genes versus disease genes in PPI and PSN

Essential genes are known to have a strong tendency to be located at the functional center of the protein interactome. However, there has been much debate about the centrality of disease genes. Disease genes from the OMIM database are reported to show higher degrees in PPI than non-disease genes (23). Recent study of complex trait-associated loci also reported that they were located at the core of PPI (24). But Goh *et al.* reported that disease genes were located not in the hub but in the periphery of protein interactome and attributed other studies’ results to the subset of disease genes that were also essential (25). They declined their initial hypothesis that disease genes might be located at the functional hub of (a certain) biological network (25). However, it seems that Goh *et al.* overlooked the possibility of disease genes to be located at the functional hub of another biological network other than PPI. After all, PPI is not the only biological network.

**Figure 2.**
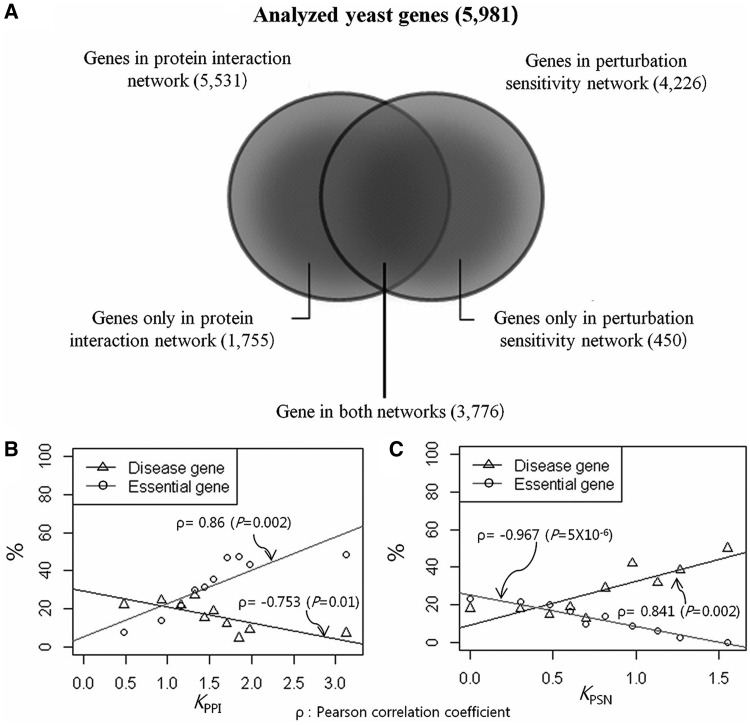
Distribution of the proportion of lethal and disease genes in biological networks. (**A**) The Venn diagram shows the distribution of yeast genes in protein interaction and perturbation networks. Distributions of lethal- and disease-gene proportions are plotted according to their degrees, *K*_PPI_ and *K*_PSN_, in (**B**) protein interaction and (**C**) perturbation networks. Lethal genes are increasingly enriched in the hub of the protein interaction network but decreasingly enriched in the hub of perturbation network. In contrast, disease genes are increasingly enriched in the hub of perturbation network but decreasingly enriched in the hub of protein interaction network. Lethal and disease genes constitute the two opposite hubs of the reciprocally scale-free biological networks, PPI and PSN, respectively.

We plotted the proportion of lethal genes in each degree bin of *K*_PPI_ and *K*_PSN_ (Figure 2B and C). Consistent with the previous study (3), the PPI core that is under high evolutionary selection pressure is increasingly enriched with lethal genes than its periphery (Figure 2B). However, the PSN core that is also under high evolutionary selection pressure is inversely enriched with lethal genes (Figure 2C). Instead, non-lethal disease genes are highly enriched in the PSN core, whereas they are very sparse in the PPI core (Figure 2C). A biological network may not be singular. Lethal genes and disease genes show inversely proportional (or reciprocal) degree distributions between the two biological networks. We propose that disease genes are located in the functional hub of PSN but not in that of PPI, which is a reciprocal network to PSN.

**Figure 3.**
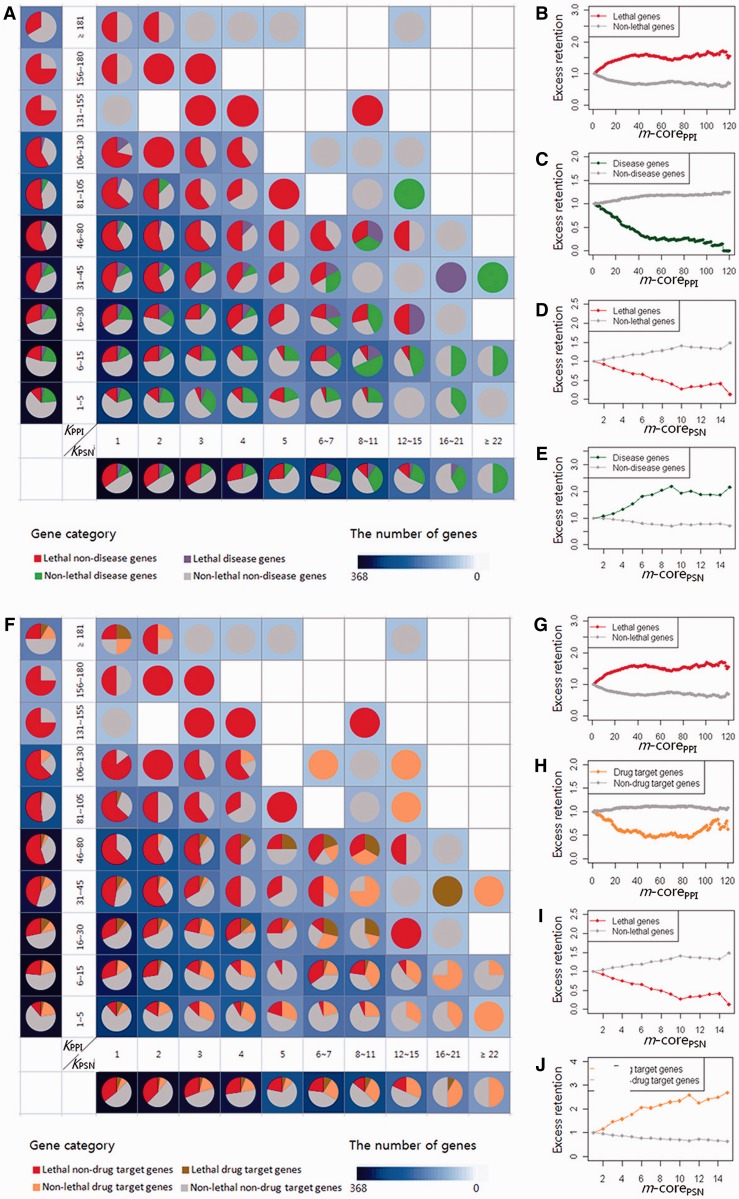
Inversely proportional degree distributions between lethal genes and disease genes or drug-target genes in the joint network of protein interaction and perturbation sensitivity. (**A**) A grid diagram that consists of pie charts that demonstrate the proportion of four groups of genes (lethal disease, lethal non-disease, non-lethal disease and non-lethal non-disease genes) at each degree bin. Pie charts at higher-degree bins of PPI (left upper diagonal) exhibit larger proportion of red (i.e. lethal non-disease) genes, whereas those at higher-degree bins of PSN (right lower diagonal) exhibit smaller proportion of red genes. On the contrary, green (i.e. disease non-lethal) genes are highly enriched at the right lower diagonal and sparse at the left upper diagonal. The marginal pie charts along with the vertical and horizontal axes also clearly demonstrate that lethal non-disease genes are increasingly enriched in the PPI core and non-lethal disease genes are enriched in the PSN core and that PPI and PSN are reciprocally intertwined. The background-color density of each cell of the grid diagram denotes the number of genes, and higher density means more genes in the cell (see Figure 1D). It demonstrates that the hubs of the protein interaction network are enriched with lethal genes but are inversely enriched with disease genes, whereas hubs of the perturbation network are enriched with disease genes but are inversely enriched with lethal genes. The excess-retention plots (b-e) of lethal genes and disease genes shown according to the *m*-cores of PPI and PSN clearly demonstrate the inversely proportional enrichments of (**B**) lethal genes in the PPI core and (**D**) disease genes in the PSN core, respectively, and (**C** and **E**) vice versa. (**F**) A grid diagram that consists of pie charts demonstrates that the hubs of protein interactome are (**G**) highly enriched with lethal genes (in red) but (**H**) inversely enriched with drug-target genes (in orange). The only exception is found with those hubs with extreme high degrees (>180). The hubs of perturbation sensitivity network are (**J**) highly enriched with drug-target genes but (**I**) inversely enriched with lethal genes. (**G**–**J**) Excess retention plots of lethal genes and drug-target genes are shown according to m-cores of the networks. Background color density of each cell denotes the number of genes.

The grid diagram that consists of pie charts in Figure 3 clearly demonstrates the reciprocal degree distribution of lethal (in red) versus disease (in green) genes in two perpendicular degree axes of the joint network (Figure 1C and D), made up of 1040 yeast genes with human homologs whose lethality and disease annotations are available. Vertices (or genes) are classified into four groups: lethal disease, lethal non-disease, non-lethal disease and non-lethal non-disease genes. Lethal non-disease genes (in red) and non-lethal disease genes (in green) are symmetrically distributed around the space diagonal *Y* = *X* axis, which means that lethal non-disease genes have high degrees in PPI but low degrees in PSN, and non-lethal disease genes are much connected in PSN but less connected in PPI.

The marginal pie-chart arrays along with the vertical and horizontal axes also clearly demonstrate that lethal non-disease genes are increasingly enriched in the PPI core and non-lethal disease genes in the PSN core and that PPI and PSN are reciprocally intertwined. Please notice that the vertical marginal pie-chart array successfully replicates Goh *et al.*’s finding that disease genes (in green and purple) are located at the functional periphery (i.e. 1 ∼ 105 PPI degrees) of the protein interactome (25). As reported by Goh *et al.*, non-lethal disease genes cannot be found at the very hub of PPI (i.e. beyond 105 degrees in our study), although they overlooked the other network of perturbation sensitivity, which is represented in the horizontal counterpart of the grid diagram.

The plots from excess-retention analysis (Figure 3B–E) clearly demonstrate that PPI hubs are enriched with lethal genes (Figure 3B), whereas PSN hubs are enriched with disease genes (Figure 3E). PPI hubs are inversely enriched with disease genes (Figure 3C), whereas PSN hubs are inversely enriched with lethal genes (Figure 3D). We suggest that disease genes are not simply located at the periphery of PPI but at the highly connected core of another network, i.e. PSN. Disease genes are closely associated with perturbation sensitivity, and highly perturbation-sensitive genes are mostly disease genes.

### Drug-target, TATA box and TFBS of PPI and PSN

Moreover, the yeast homologs of drug-target genes extracted from the DrugBank database (http://www.drugbank.ca) are similarly located at the core of PSN and in the periphery of PPI, although there is an exceptional enrichment of drug-target genes among the PPI hubs with very high degrees (Figure 3F–J and Supplementary Figure S1). PSN hubs are also highly enriched with more transcription factors and TATA boxes in their promoter region (Supplementary Figures S2 and S3).

### Functional annotation analysis of PPI and PSN

**Figure 4.**
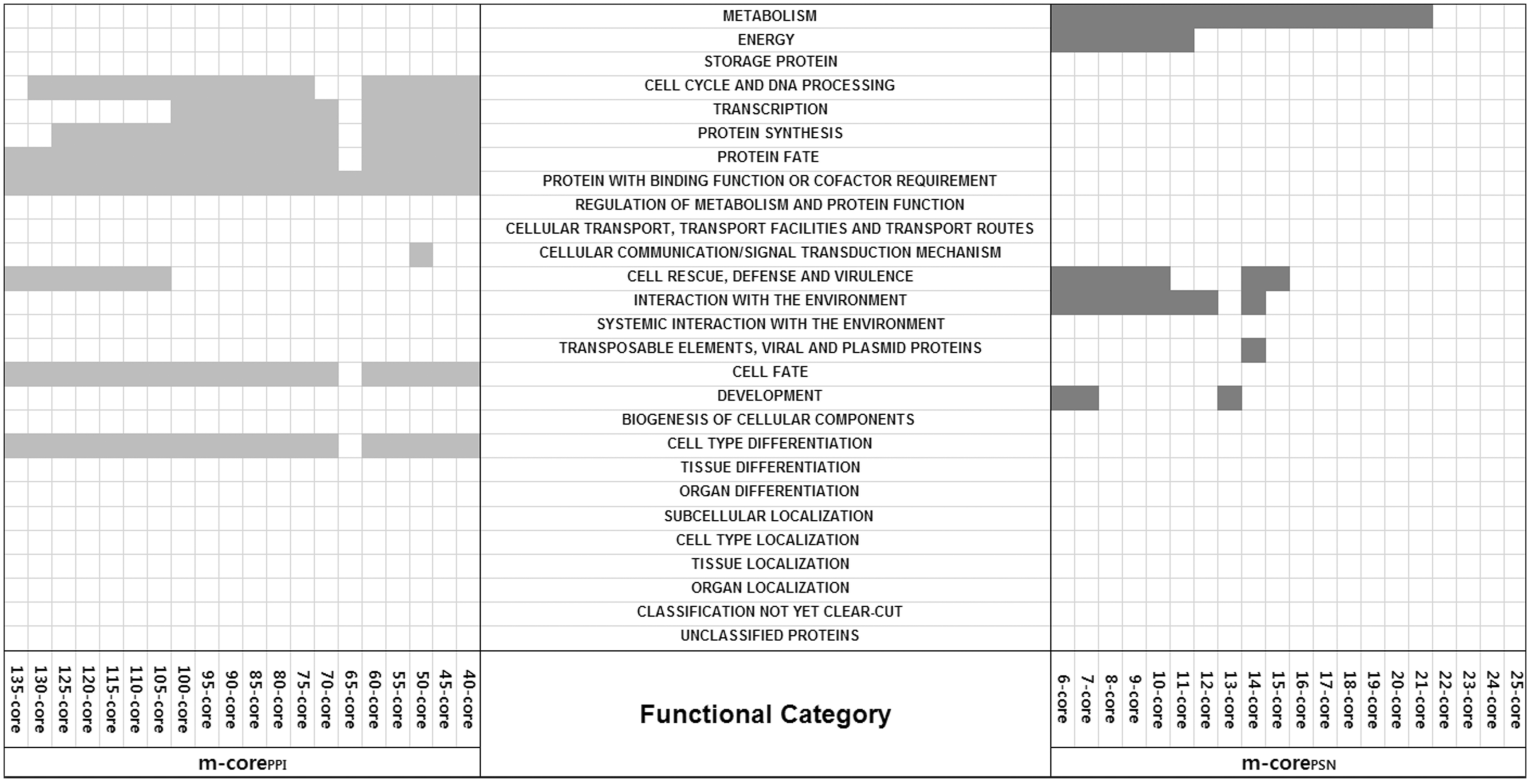
The distribution of functional annotation categories (FDR-adjusted *P* < 0.01, see ‘Materials and Methods’ section) in each group of *m*-core_PSN_ (m ≥ 6) and *m*-core_PPI_ (m ≥ 40). If a functional category is significantly overrepresented in a group, then the corresponding rectangle is colored in orange for the protein interactome and in blue for the perturbation sensitivity network. The hubs of the protein interactome are enriched with ‘cell cycle and DNA processing’, ‘transcription’, ‘protein synthesis’, ‘protein fate’, ‘protein with binding function or cofactor requirement’, ‘cell fate’ and ‘cell type differentiation’, whereas those of the perturbation sensitivity network are enriched with metabolism, ‘cell rescue’, ‘defense and virulence’, ‘interaction with the environment’, ‘transposable elements’ and ‘development’. Two hubs have mutually exclusive functional annotations.

Gene Ontology-based functional annotation enrichment analysis with the online tool of the MIPS database (http://mips.gsf.de/proj/funcatDB) demonstrates a mutually exclusive enrichment pattern between the hubs of PPI and PSN. PPI hubs are enriched with essential cellular processes, whereas PSN hubs are enriched with environmental interactions (Figure 4, FDR-adjusted *P* < 0.01). Although the PPI hubs are enriched with ‘cell cycle and DNA processing’, ‘transcription’, ‘protein synthesis’, ‘protein fate’, ‘protein with binding function or cofactor requirement’, ‘cell fate’ and ‘cell type differentiation’, the PSN hubs are enriched with ‘metabolism’, ‘cell rescue’, ‘defense and virulence’, ‘interaction with the environment’, ‘transposable elements’ and ‘development’.

### Network visualization of PPI and PSN

Supplementary Figure S4 visualizes the individual (Supplementary Figures S4A–D) and joint (Supplementary Figure S4E–H) networks in three-dimensional space with edges visualized (Supplementary Figure S4A, C and E) or omitted (Supplementary Figure S4B, D and F). The edges of the joint network are made up of two distinct edge sets: the undirected edge set of PPI and the directed edge set of PSN. It is a mixed graph *G* = (*V*, *E*_PPI_ and *E*_PSN_), and the degree of vertex *V* is a vector of two elements (*K*_PPI_, *K*_PSN_). The proportion of the shared edges (in black, Supplementary Figure S4G) between two networks is only 0.3% (*n* = 98) of the 35 908 edges of the whole joint network. The connectivities of the joint network are not independent but inversely proportional to each other. The perturbation network is the disease network, whereas the protein interaction network is the essentiality network, and the two reciprocal and separate halves seem to constitute a more comprehensive model for the yeast gene network (Supplementary Figure S4E–H).

## DISCUSSION

In a biological sense, the cellular transcriptome has been suggested to be bipolar (‘internal growth’ versus ‘external stress response’). Living cells have to balance two important biological conditions. In a nutrient-rich condition, yeasts activate a transcriptional program to spend most of their energy and metabolic substrates for maximal proliferation, although they become labile to external stress. In a nutrient-deprived environment, however, they enter a quiescent state and a reciprocally distinct set of transcriptional program is recruited for high stress resistance and the transcriptome for proliferation and growth is suppressed (6,26). The internal growth signal is to lethality as the external stress response signaling is to disease. The two programs are equally important, and living cells with a defect in growth program do not ‘survive’, whereas those with a dysfunctional stress response do not ‘thrive’ in environmental stress.

One limitation of the current study is the comparability between PPI and PSN, which are physical interaction and functional responsiveness networks, respectively, of different kinds. In graph theory, however, the two networks can be represented as a mixed graph with two different edge types. Although the edges come from different (i.e. physical and relational) classes, we were able to demonstrate that the hubs of both networks uniformly showed evolutionary conservations (Figure 1E and F). The fact that the evolutionary selection pressure, which is a fundamental process working on all biological systems, has consistently impacted both networks suggests that the two networks of different kinds may be considered together at the systems level of biological understanding.

Physical interaction of genes may restrain their functional interaction and vice versa. The PPI hub genes are directly connected to many genes, and too much ‘fluctuation’ of them beyond a certain degree may severely disrupt the biological system (or the cell). Thus, many of them are highly lethal genes. Both PPI and PSN hubs may receive many inputs. PPI hubs may respond to a variety of inputs in a mildly modulated fashion, impacting many genes. On the other hand, PSN hubs may show a strong response to specific sets of signals but the impact may be restrained to limited sets of genes. PSN hubs have many regulatory genes like transcription factors (see Supplementary File of PPI and PSN core gene lists). A singular network may not be sufficient for autonomous biological systems.

We considered the genes that were most ‘perturbed’ to be PSN hubs, whereas Rung *et al.* (9) considered the most ‘perturbing’ genes to be hubs of their disruption network. They found that the most ‘perturbing’ genes were mostly ‘regulatory genes’ like transcription factors. The most severely perturbing genes, however, must be the essential genes themselves because disturbed essential genes may cause complete cell death, hence impacting all genes. Therefore, bias to non-lethal genes is inevitable. In contrast, the definition of the most perturbed genes is unbiased because the effect of lethal mutation can equally be applied to all genes. Because there are only 273 genes that can be mutated without lethal effect out of the whole yeast genes in the original experiment, we did not further characterize their lethal disease relationship for the ‘perturbing’ genes whose deletion affects the expression of other genes.

Unlike artificial networks, biological networks do not grow indefinitely. The hitherto ever-growing singular network model lacks a way to model the self-autonomous and self-constrained features of actual biological systems. Network growth by preferential attachment in physical interaction may be constrained by the connectivity in transcriptional interaction and vice versa. The negative correlation seems to organize the joint network into two reciprocal cores, where preferential attachments of nodes are self-constrained between lethality versus disease, growth versus stress response or survival versus thrival. Integrating two scale-free networks that are reciprocal to each other but work in concert for the same goal might provide a realistic model of biological systems. Life is maintained self-autonomously by the unity of opposites, yin and yang, or ‘conjunctio oppositorum’.

## SUPPLEMENTARY DATA

Supplementary Data are available at NAR Online.

## FUNDING

The basic science research program through the National Research Foundation of Korea (NRF); Korea government (MSIP) [2013-005540]; Ministry of Health & Welfare [A120300]. Funding for open access charge: National Research Foundation of Korea (NRF).

*Conflict of interest statement*. None declared.

## Supplementary Material

Supplementary Data

Supplementary Data
