# Supplementary material for: Yin and Yang of disease genes and death genes between reciprocally scale-free biological networks
Source: Nucleic Acids Res. 2013 Aug 9;41(20):9209–17. doi: 10.1093/nar/gkt683 (PMC3814386; doi:10.1093/nar/gkt683)
Supplement: Supplementary Data [file supp_41_20_9209_v2_index.html]

Yin and Yang of disease genes and death genes between reciprocally scale-free biological networks — Yin and Yang of disease genes and death genes between reciprocally scale-free biological networks — Supplementary Data 

# Yin and Yang of disease genes and death genes between reciprocally scale-free biological networks

## Supplementary Data

files

**Files in this Data Supplement:**

- Supplementary Data - doc file
