# Supplementary material for: Yin and Yang of disease genes and death genes between reciprocally scale-free biological networks
Source: Nucleic Acids Res. 2013 Aug 9;41(20):9209–17. doi: 10.1093/nar/gkt683 (PMC3814386; doi:10.1093/nar/gkt683)
Supplement: Supplementary Data [file supp_gkt683_Supplement_gkt683.doc]

Supplementary Information

**Yin and Yang of disease genes and death genes between reciprocally scale-free biological networks**

Hyun Wook Han **^*^**, Jung Hun Ohn**^*^**, Jisook Moon**^†^** , Ju Han Kim**^†^**

*equally contributed

**^†^** To whom correspondence should be addressed.

Email: [juhan@snu.ac.kr](mailto:juhan@snu.ac.kr); [jmoon@cha.ac.kr](mailto:jmoon@cha.ac.kr)

**This PDF file includes:**

Supplementary Figures S1 to S4 and Table S1

**SUPPLEMENTALARY FIGURES**


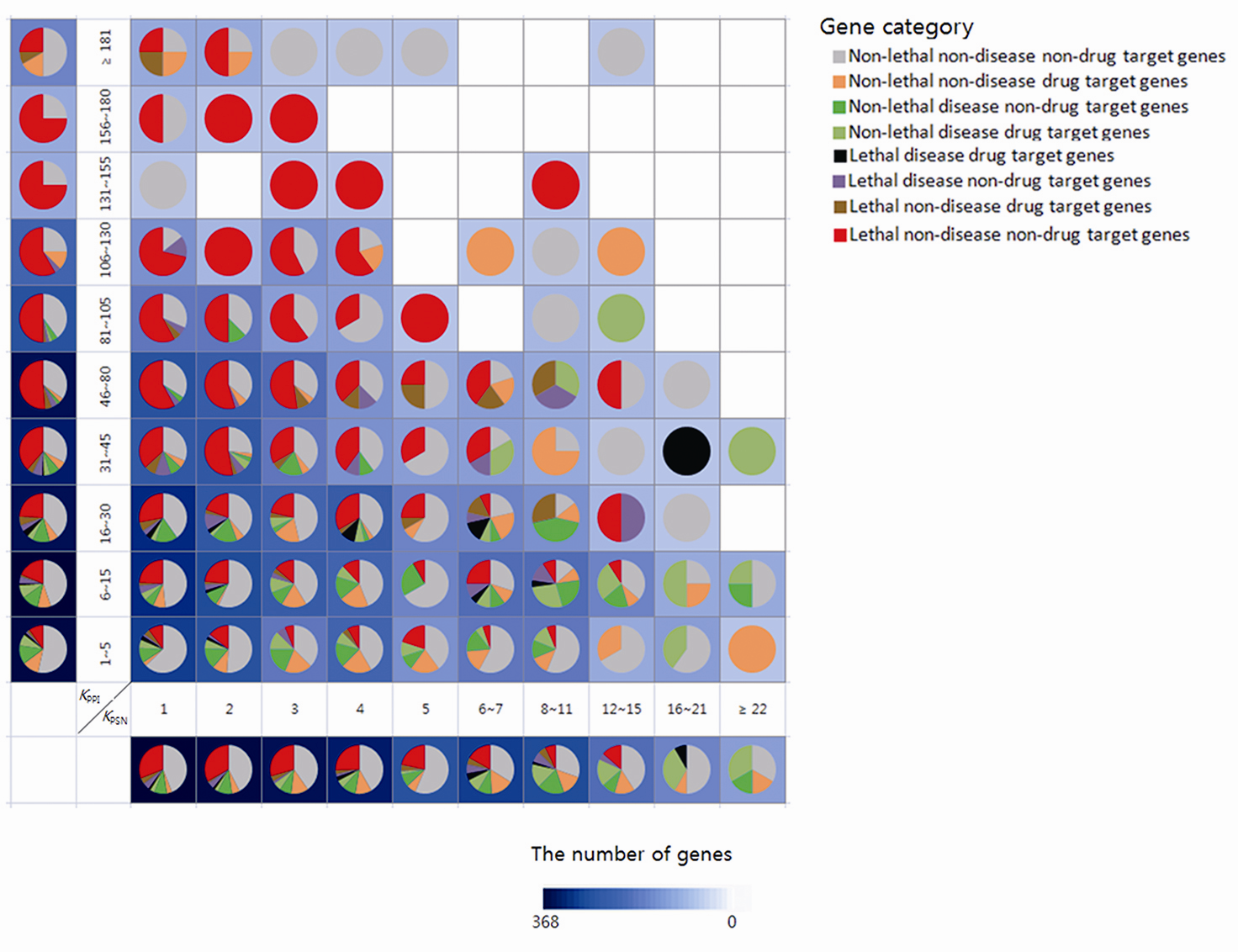


**Supplementary Figure S1.** Distribution of lethal, disease, and drug-target genes of 1,040 human homologues in the joint network. The hubs of perturbation sensitivity networks are enriched not only with disease genes but also with drug-target genes (> 180) whereas the hubs of protein interactome are enriched with lethal genes except for those with extreme high degrees. Background color density of each cell denotes the number of genes.

**
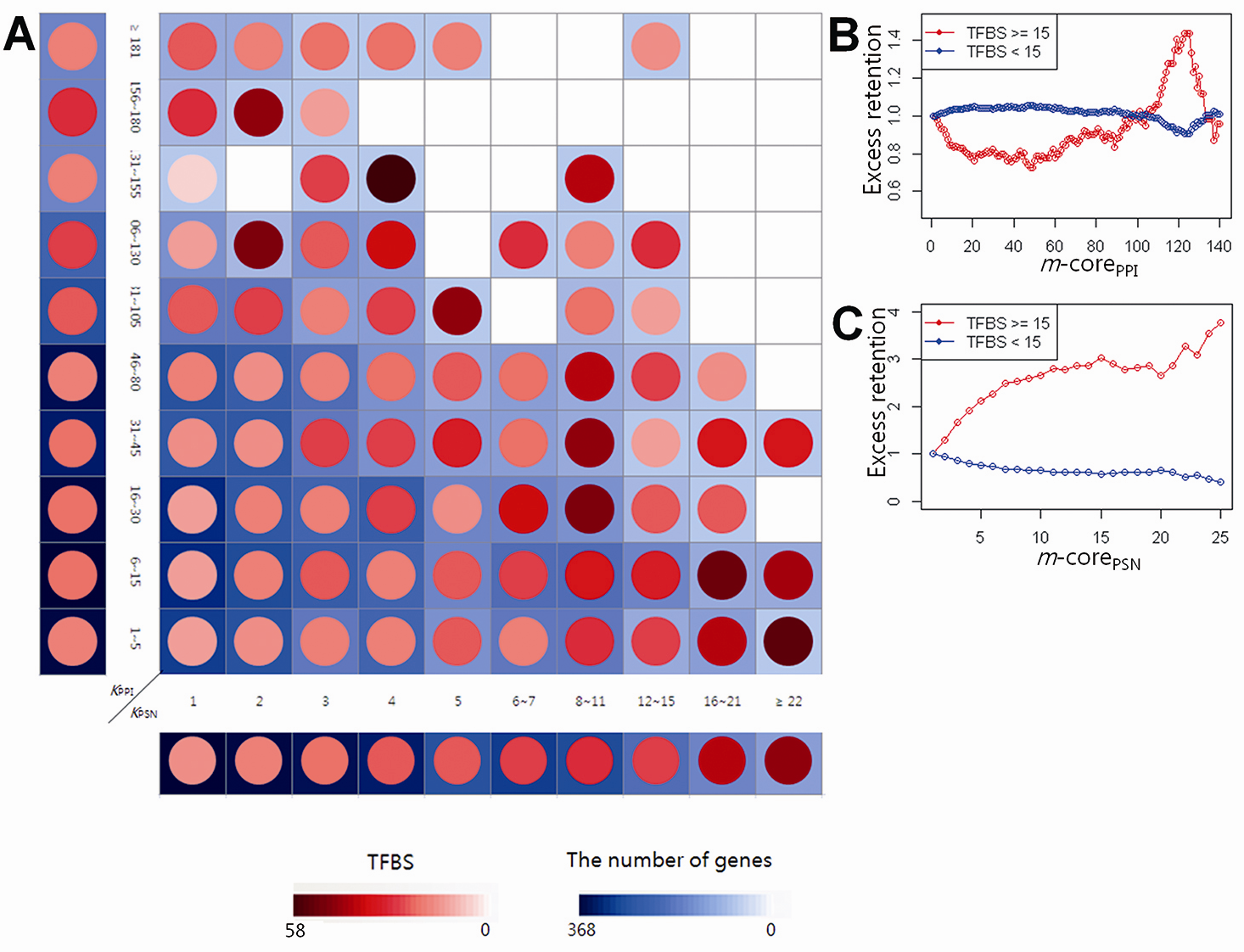
**

**Supplementary Figure S2.** Distribution of the numbers of the transcription factor binding sites (TFBSs) of 3,776 yeast genes in the joint network. (A) The horizontal marginal array of circles next to a grid diagram demonstrates that the hubs of perturbation sensitivity network hubs are highly enriched with TFBSs. (B) Excess retention plot of protein interactome does not show a distinct enrichment tendency according to *m*-core degrees but those hubs with very high degrees between 156 to 180 show strong enrichment. (C) Excess retention plot of perturbation sensitivity network shows strong enrichment tendency of TFBS according to *m*-core degrees. Genes are divided into two groups as low (TFBS ≥ 15) and high (TFBS < 15) numbers of TFBSs in the two excess retention plots. Background color density of each cell denotes the number of genes.


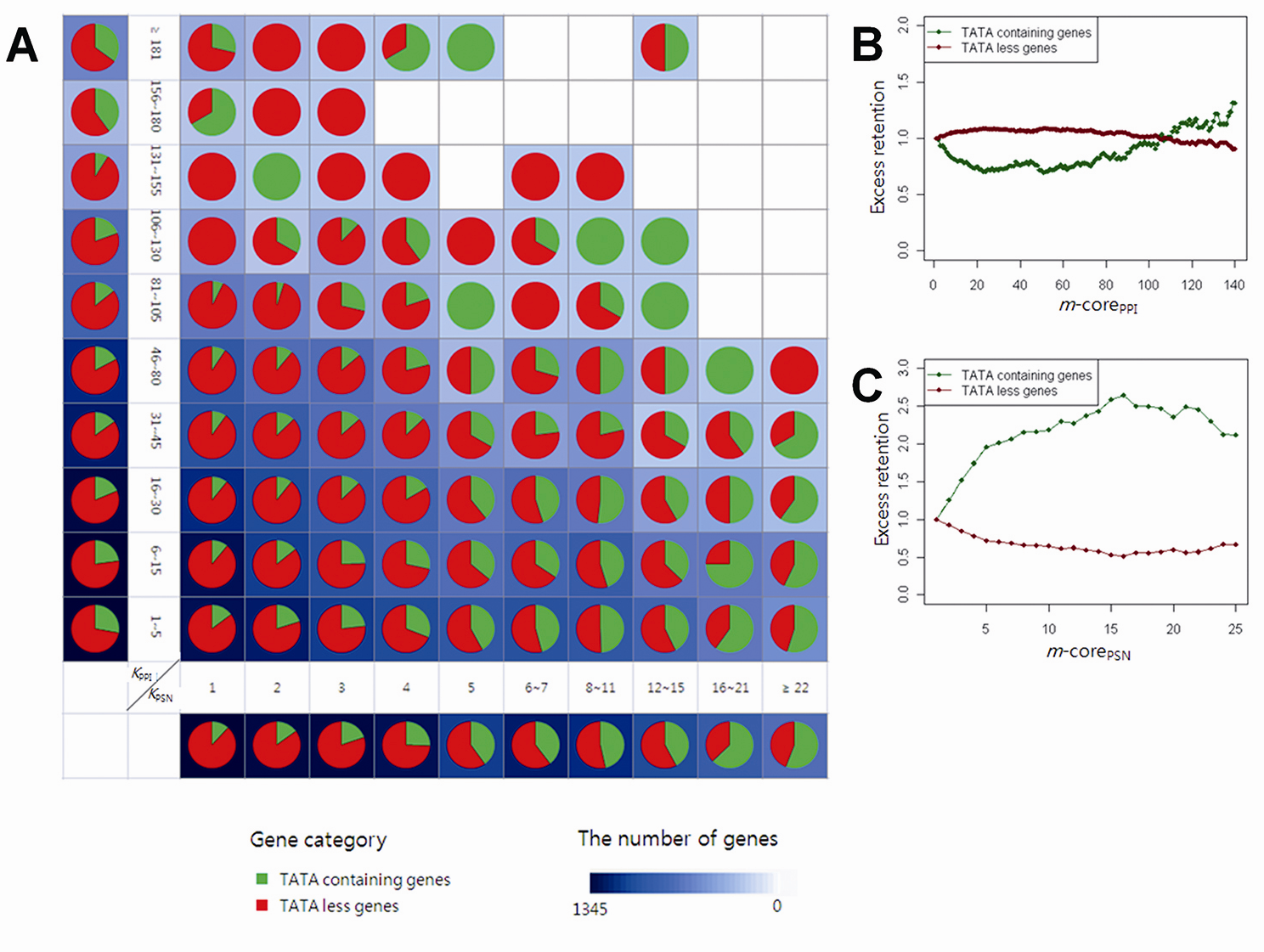


**Supplementary Figure S3.** Distribution of TATA-containing genes in the joint network of the 3,737 yeast genes. (A) A grid diagram that consists of pie charts demonstrates that the hubs of perturbation sensitivity network are enriched with TATA-containing genes (in green) as shown in the horizontal marginal array of pie charts. The hubs of protein interactome, however, do not show a distinct enrichment pattern. Excess retention plots of TATA-containing and TATA-less genes in (B) the protein interactome and (C) the perturbation sensitivity network are shown to *m*-cores. Background color density of each cell denotes the number of genes.

**
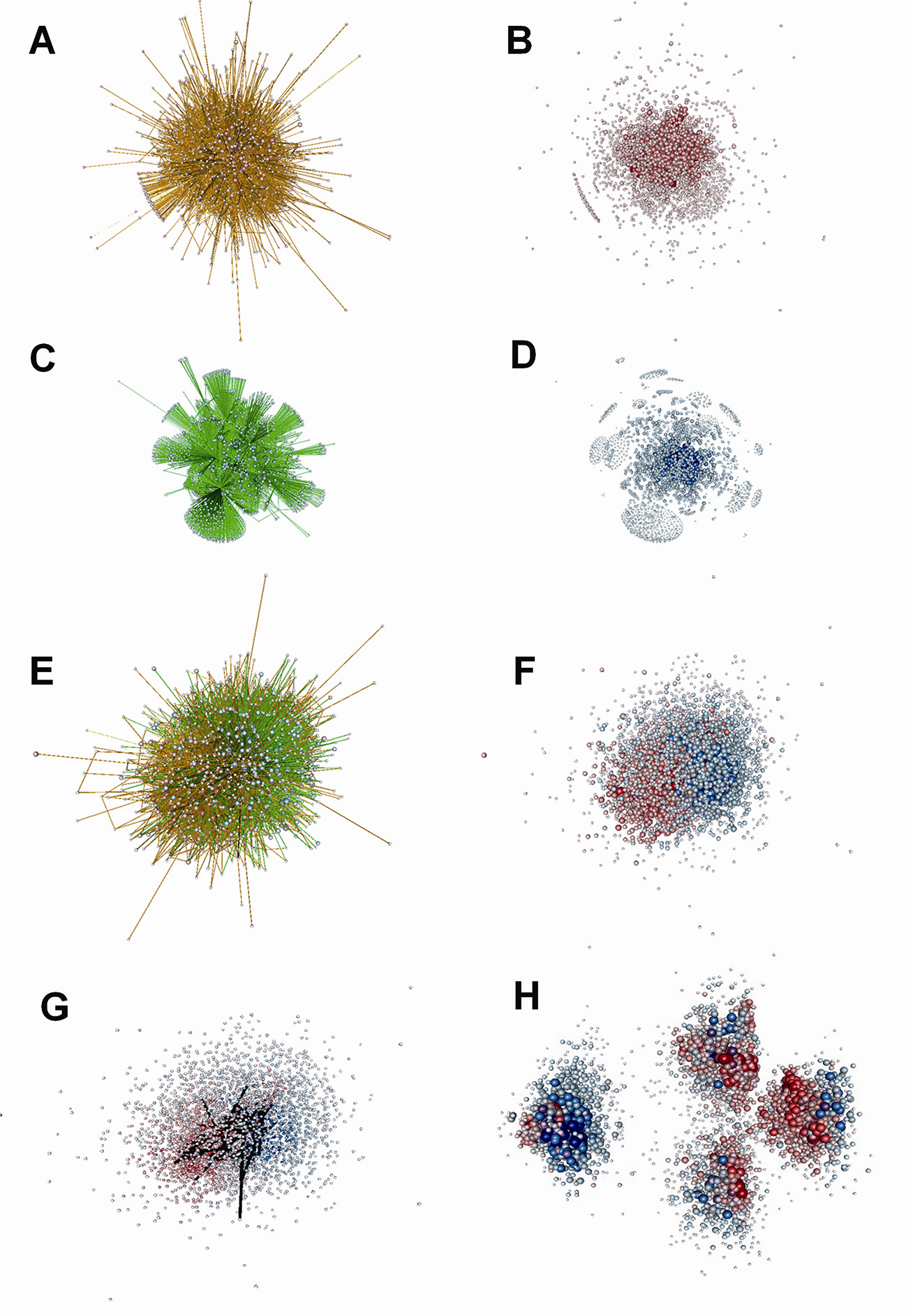
**

**Supplementary Figure S4.** Three-dimensional visualization of biological networks. The protein interaction network is visualized with 3,679 nodes (in blue beads) and 26,088 edges (in green lines) with edges (A) shown and (B) omitted. The perturbation sensitivity network is visualized with 3,392 nodes (in red beads) and 9,820 edges (in yellow lines) with edges (C) shown and (D) omitted. Joint network is visualized with 3,765 nodes and 35,908 edges with edges (E) shown and (F) omitted. (G) The dual edges representing both protein-interaction and perturbation links in the joint network are visualized (in black lines). Only 0.3% (=98/35,908) of the edges are found in both networks (H) The Joint network with omitted edges is split into four equally shaped tetrahedral pieces and unfolded to visualize the internal structure of the joint network.

**Supplementary Table S1**. The list of abbreviations.

| **List of Abbreviations** | **Full names** |
| --- | --- |
| **PPI** | Protein-protein interaction network |
| **PSN** | Perturbation sensitivity network |
| **TFBS** | Transcription Factor Binding Sites |
| **dN/dS** | Evolutionary rate |
| **dN** | The number of non-synonymous substitutions per non-synonymous site |
| **dS** | The number of synonymous substitutions per synonymous site |
| **CAI** | Codon Adaptation Index |
| **K_PPI_** | Node degree in protein-protein interaction network |
| **K_PSN_** | Node degree in perturbation sensitivity network |
